# Supplementary material for: Causal Relationship between Aging and Anorexia Nervosa: A White-Matter-Microstructure-Mediated Mendelian Randomization Analysis
Source: Biomedicines. 2024 Aug 16;12(8):1874. doi: 10.3390/biomedicines12081874 (PMC11351342; doi:10.3390/biomedicines12081874)
Supplement: Supplementary file 1 [file biomedicines-12-01874-s001.zip › Supplementary Figure/Figure S8/Leaveoneout plot of MR analysis for TL to ubm-b-2020.pdf]

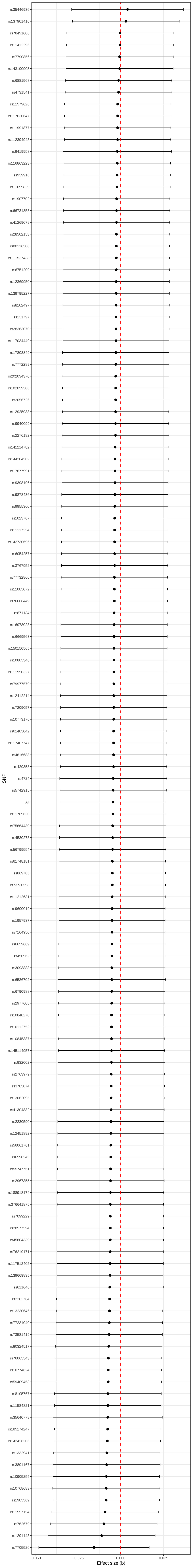
rs35446936
rs137901416
rs78491606
rs11412296
rs7790856
rs143190905
rs6881568
rs4731541
rs11579626
rs117630647
rs11991877
rs112394943
rs9419958
rs116863223
rs939916
rs11699829
rs1907702
rs66731853
rs41269079
rs28502153
rs80116508
rs111527438
rs6751209
rs12369950
rs139795227
rs8102497
rs1317997
rs28363070
rs117034449
rs17803849
rs7772289
rs202034370
rs182059586
rs2056726
rs12925933
rs9940099
rs2276182
rs141214782
rs144204502
rs17677991
rs9398196
rs9878436
rs9955360
rs1023767
rs11117354
rs142730696
rs6054257
rs3767952
rs77732866
rs11085072
rs76664449
rs871134
rs16978028
rs6669563
rs150150565
rs10805346
rs111950327
rs79977579
rs12412214
rs7209057
rs10773176
rs61405042
rs117407747
rs4616688
rs429358
rs4724
rs5742915
All
rs11769630
rs75664430
rs4530278
rs56799554
rs61748181
rs869785
rs73730598
rs11212631
rs9600019
rs1957937
rs7164950
rs6659669
rs450962
rs3093888
rs6536702
rs6790988
rs2977608
rs10840270
rs10112752
rs10845387
rs145114957
rs932002
rs2763979
rs3785074
rs13062095
rs41304832
rs2230590
rs12451892
rs56061761
rs6590343
rs55747751
rs2967355
rs188918174
rs376641875
rs7099229
rs28577594
rs45604339
rs76219171
rs117512405
rs139669835
rs611646
rs2282764
rs13230646
rs77231040
rs73581419
rs80324517
rs76065543
rs10774624
rs59409453
rs8105767
rs11584821
rs35640778
rs185174247
rs142426306
rs1332941
rs3891167
rs10905255
rs10768683
rs1985369
rs11557154
rs762679
rs1291143
rs7705526
SNP
-0.050
-0.025
0.000
0.025
Effect size (b)
